# Supplementary material for: Screening of polyhydroxyalkanoate-producing bacteria and PhaC-encoding genes in two hypersaline microbial mats from Guerrero Negro, Baja California Sur, Mexico
Source: PeerJ. 2018 May 7;6:e4780. doi: 10.7717/peerj.4780 (PMC5944434; doi:10.7717/peerj.4780)
Supplement: Supplemental Information 1 — phaC1 sequences alignment of 13 Pseudomonas species where primers phaC1F1, phaC1F2, phaC1R1, and phaC1R2 were designed (Table 1). Zones for primers design are shown in red. phaC sequences used in the alignment were as follows: P_a = Pseudomonas alkylphenolia GenBank No. CP009048; P_e = Pseudomonas extremaustralis GenBank No. FN435843; P_f = Pseudomonas fulva GenBank No. CP002727; P_m = Pseudomonas mendocina GenBank No. DQ316602; P_n = Pseudomonas nitroreducens GenBank No. AF336849; P_r = Pseudomonas resinovorans GenBank No. AP013068; P_s = Pseudomonas stutzeri GenBank No. AY278219; P_sp = Pseudomonas sp. GenBank No. KJ169572; P_p1 = Pseudomonas pseudoalcaligenes GenBank No. LK391695; P_d = Pseudomonas denitrificans GenBank No. CP004143; P_k = Pseudomonas knackmussii GenBank No. HG322950; P_c = Pseudomonas chlororaphis GenBank No. CP011110; P_p2 = Pseudomonas putida GenBank No. CP010979. [file peerj-06-4780-s001.docx]

**PhaC1F2 5’ SATCAACCTGATGACCGA 3’**

351

P_a ACATCAGTCG CGGTCACTTC GTCATCAACC TGATGACCGA AGCCATGGCC CCGACCAACA

P_e ACATCAGCCG CGCCCACTTC GTGATCAACC TGATGACCGA AGCCATGGCC CCCACCAACA

P_f ACGTCAGCCG CGGCCACTTC ATCATCAACC TGCTGACCGA GGCGATGGCG CCGAGCAACA

P_m ACGCCAGCCG CGGCCACTTC GTGATCAACC TGATGACCGA AGCCATGGCA CCCTCCAACA

P_n ACGCCAGCCG CGGCCATTTC GTGATCAACC TGATGACCGA AGCCATGGCG CCTTCCAACA

P_r ACAGCAGCCG CGGCCACTTC GTCATCAACC TGATGACCGA GGCCATGGCG CCCACCAACA

P_s ACGCCAGCCG CGGCCATTTC GTGATCAACC TGATGACCGA GGCCATGGCA CCAAGCAACA

P_sp ACGCCAGTCG CGGCCACTTC GTCATCAACC TGATGACCGA AGCCATGGCT CCATCCAACA

P_p1 ACGCCAGTCG CGGCCACTTC GTGATCAACC TGATGACCGA GGCCATGGCG CCGTCCAACA

P_d ACATCAGTCG CGGCCAGTTC GTCATCAACC TGATGACCGA AGCGATGGCA CCGACCAATA

P_k ACATCAGCCG CGGGCAGTTC GTCATCAACC TGATGACCGA AGCCATGGCG CCAACCAACA

P_c ACCAGGCCCG CGCGCACTTT GTCTTCAGCC TGCTCAACGA TGCCGTCGCC CCCTCCAACA

P_p2 ACCGCGCCCG CGCACATTTT CTGTTCGGCA TGATCAATGA CGCCCTGGCA CCGAGCAACT

411

P_a GCGCGGCCAA CCCGGCGGCG GTCAAACGTT TCTTTGAAAC CGGCGGCAAA AGCCTGCTCG

P_e GCGCGGCCAA CCCGGCGGCG GTCAAGCGCT TCTTTGAAAC CGGCGGCAAA AGCCTGCTCG

P_f GCCTGGCCAA CCCGGCAGCG CTCAAACGGA TCTTCGACAC CGGCGGCAAG AGTCTGCTCG

P_m GCATGGCCAA CCCGGCAGCG GTCAAACGTT TCTTCGAAAC CGGCGGCAAG AGCCTGCTCG

P_n GCATGGCCAA TCCGGCGGCG GTCAAGCGCT TCTTCGAAAC CGGCGGCAAG AGCCTGCTCG

P_r GCATGGCCAA CCCGGCTGCG GTGAAGCGCT TCTTCGAGAC CGGCGGCAAG AGCCTGCTGG

P_s GCATGGCCAA CCCGGCCGCG GTCAAACGCT TCTTCGAGAC CGGCGGCAAG AGCCTGCTCG

P_sp GCATGGCCAA CCCGGCCGCG GTCAAACGCT TCTTCGAAAC CGGCGGCAAG AGCCTGCTCG

P_p1 GCATGGCCAA CCCGGCCGCG GTCAAACGCT TCTTCGAGAC CGGCGGCAAG AGCCTGCTCG

P_d CGCTGTCCAA CCCGGCGGCG GTCAAACGCT TCTTCGAGAC CGGCGGCAAG AGCCTGCTCG

P_k CCCTGAGCAA CCCGGCAGCG GTCAAGCGCT TCTTCGAGAC CGGCGGCAAG AGCCTGCTCG

P_c CCCTGCTCAA CCCGCTGGCG ATCAAGGAAA TATTCAACTC CGGCGGCAAC AGCGTGATCC

P_p2 CGCTGCTCAA CCCGCTGGCG GTCAAAGAGC TGCTGAACAC GGGCGGGCAA AGCCTGGTGC

471

P_a ACGGCCTGTC GAACCTGGCC AAGGACATGG TCCACAATGG CGGCATGCCC AGCCAGGTGA

P_e ATGGCCTGTC CCACCTGGCC AAGGACATGG TGCATAACGG CGGCATGCCG AGCCAGGTCA

P_f ATGGCCTTTC GCACATGGCC AAGGATCTGC TGAACAACGG TGGCATGCCC AGCCAGGTGG

P_m ACGGCCTGTC GCACCTGGCC AAGGACATGG TGCACAACGG CGGCATGCCG AGTCAGGTGA

P_n ATGGTCTCTC GCACCTGGCC AAGGACATGG TGCACAACGG TGGCATGCCC AGCCAGGTGA

P_r ACGGCCTTTC CCACCTGGCC AAGGACCTGG TCAACAACGG CGGCATGCCG AGCCAGGTGA

P_s ACGGCATGTC GCACCTGGCC AAGGACATGA TCAACAACGG CGGCATGCCC AGCCAGGTCA

P_sp ATGGTCTCTC CCACCTGGCC AAGGACATGG TGCACAACGG CGGCATGCCC AGCCAGGTGA

P_p1 ACGGTCTGTC GCACCTGGCC AAGGACATGG TGCACAACGG AGGCATGCCC AGCCAGGTGA

P_d ACGGCCTCAC CCACCTGGCC AAGGACCTGG TGAACAACGG CGGCATGCCC AGCCAGGTGA

P_k ACGGCCTCAG CCACCTTGCC AAGGACCTGG TGAACAACGG CGGCATGCCC AGCCAGGTGA

P_c GCGGCATCGG TCATCTGGTG GACGACCTGC TGCACAACAA CGGCCTGCCC AGCCAGGTCA

P_p2 GGGGCGTGGC CCACTTGCTT GACGACCTGC GCCACAATGA CGGCCTGCCG CGCCAGGTGG

531

P_a ACATGGAGGC CTTCGAGGTC GGCAAGAACC TGGGCACCAG CGAAGGCGCA GTGGTGTTTC

P_e ATATGAAAGC GTTCGAGGTG GGCAAGACCC TGGGCACCAC CGAAGGCGCG GTGGTGTTTC

P_f ACATGAAGGC CTTCGAAGTG GGCCGCAACC TGGCCACCAC CGAAGGCGCC GTGGTGTTTC

P_m ATATGGAGGC CTTCGAGGTC GGCAAGAACC TGGCCACCAC CGACGGCGCC GTGGTGTTTC

P_n ACATGGAGGC CTTCGAGGTT GGCAAGAATC TGGCCACCAC CGAGGGCGCC GTGGTGTTCC

P_r ACATGGACGC CTTCGAGGTC GGCAAGAACC TGGCCACCAC CGAAGGCGCG GTGGTGTACC

P_s ACATGGCCGC CTTCGAAGTA GGCAAGAACC TGGCCACCAC CGAGGGCGCC GTGGTTTTCC

P_sp ACATGGAGGC CTTCGAGGTC GGCAAGAACC TGGCCACCAC CGACGGCGCC GTGGTGTTTC

P_p1 ACATGGAAGC CTTCGAGGTC GGCAAGAACC TGGCCACCAC CGACGGCGCC GTGGTGTTTC

P_d ACATGGACGC CTTCGAGGTC GGCAAGAACC TGGCCCTGAC CGAAGGCGCA GTGGTGTTCC

P_k ACATGGACGC CTTCGAGGTC GGCAAGAACC TCGCCACCAC CGAAGGCGCC GTGGTGTTCC

P_c CCAAACAGGC TTTCGAGGTC GGCCGCAACC TGGCCACCAC CCCCGGCTCG GTGGTGTTTC

P_p2 ATGAACGCGC CTTCGAGGTA GGTGGCAGCC TGGCGGCAAC GCCCGGCGCG GTGGTGTTTC

**PhaC1F1 5’ TGGARCTGATCCAGTAC 3’**

591

P_a GCAACGACGT GCTGGAACTG ATCCAGTACC GGCCGATCAC CGAACAGGTG CATGCCCGCC

P_e GCAACGATGT GCTGGAGCTG ATCCAGTACA TGCCGATCAC CGAGCAGGTG CACGAACGCC

P_f GCAACGAGGT GCTGGAGCTG ATCCAGTACA CGCCGGTCAC CGAGCAGGTC TACAAACGCC

P_m GCAACGATGT GCTGGAGCTG ATCCAGTACA AGCCGATCAC CGAGAGCGTG CATGAGCGCC

P_n GCAACGACGT GCTGGAGCTG ATCCAGTACA AACCGATCAC CGAAAGCGTG CACGAGCGCC

P_r GCAACGACGT ACTGGAGCTG ATCCAGTACA AACCCATCAC CGAGAGCGTG CACGAGCGCC

P_s GCAACGACGT GCTGGAGCTG ATCCAGTACA AGCCGATCAC CGAGAGCGTG CACGAGCGCC

P_sp GCAACGACGT GCTGGAGCTG ATCCAGTACA AGCCGATCAC CGAGAGCGTG CACGAGCGTC

P_p1 GCAACGACGT GCTGGAGCTG ATTCAGTACA AGCCGATCAC CGAGAGCGTG CACGAGCGCC

P_d GCAACGATGT GCTGGAACTG ATCCAGTACA AGCCGATCAC CGAGAGCGTG CACGAGCGCC

P_k GCAACGACGT GCTGGAACTG ATCCAGTACA AGCCGATCAC CGAGAGCGTG CACGAACGCC

P_c GCAACGAACT GCTGGAACTG ATCCAGTACA AGCCGATGAG CGAAAAACAG TACGCCCGGC

P_p2 GCAACGAGCT GCTGGAGTTG ATCCAGTACA AGCCGATGAG TGAAAAACAG CACGCCAGGC

651

P_a CGCTGCTGGT GGTGCCGCCA CAGATCAACA AGTTCTATGT CTTCGACCTG AGCCCGGAG-

P_e CGCTGCTGGT GGTCCCGCCG CAGATCAACA AATTCTATGT CTTCGACCTG AGCCCGGAT-

P_f CGCTGCTGGT GGTGCCGCCA CAGATCAACA AGTACTACGT ATTCGATTTG TCGCCGGAA-

P_m CGTTGCTAGT GGTGCCGCCG CAGATCAACA AGTTCTATGT CTTCGACCTG TCGCCGGAC-

P_n CACTGCTGGT GGTGCCGCCG CAGATCAACA AGTTCTACGT CTTCGACCTG TCGCCGGAC-

P_r CGCTGCTGGT GGTGCCGCCG CAGATCAACA AGTTCTACGT CTTCGACCTG TCACCGGAA-

P_s CGCTGCTGGT GGTGCCGCCG CAGATCAACA AGTTCTACGT GTTCGACCTG TCGCCGGAC-

P_sp CGCTGCTGGT GGTGCCACCG CAGATCAACA AGTTCTATGT ATTCGACCTG TCACCCGAC-

P_p1 CGCTGCTGGT GGTGCCACCG CAGATCAACA AGTTCTACGT CTTCGACCTG TCGCCGGAG-

P_d CGCTGCTGGT GGTGCCGCCG CAGATCAACA AGTTCTACGT CTTCGACCTG TCGCCGGAG-

P_k CGCTGCTGGT GGTGCCGCCG CAGATCAACA AGTTCTACGT GTTCGACCTG TCGCCGGAG-

P_c CAGCCCGACC AACAGCTTCG TTCAATTCGC CCTGAAGAAC GGCCT-GCAG ACCTTCATGA

P_p2 CCGTGCTGGT GGTGCCACCG CAAATCAACA AGTTCTATAT CTTCGACATG CAG-GCGCAC

711

P_a AAGAGCCTGG CGCGCTTCTG CCTGCGC-TC CAATGTACAG ACCTTCATCG TCAGCTGGCG

P_e AAGAGCCTGG CGCGCTTCTG CCTGCGC-AA CAGCCAGCAG ACCTTTATCG TCAGTTGGCG

P_f AAAAGCCTGG CGCGCTTTCT GTTGCGCAGC CAGGT-GCAG ACCTTCATCG TCAGCTGGCG

P_m AAGAGCCTGG CGCGCTTCCT CCTGCGCAGC CAGGT-GCAG ACCTTCGTGG TCAGCTGGCG

P_n AAGAGCCTGG CGCGCTTCCT CCTGCGCAGC CAGGT-GCAG ACCTTCGTGG TCAGCTGGCG

P_r AAGAGCCTGG CGCGCTTCTG CCTGCGCAAT GGCCT-GCAG ACTTTCATCG TCAGCTGGCG

P_s AAGAGCCTGG CGCGCTTCCT GCTGCGCAGC CAGGT-GCAG ACCTTCGTGG TCAGCTGGCG

P_sp AAGAGCCTGG CGCGCTTCCT GTTGCGCAGC CAGGT-GCAG ACCTTCGTGG TCAGCTGGCG

P_p1 AAGAGCCTGG CGCGCTTCCT GTTGCGCAGC CAGGT-GCAG ACCTTCGTGG TCAGCTGGCG

P_d AAGAGCCTGG CGCGCTTCTG CCTGCGCAAC AGCGT-GCAG ACCTTCATCA TCAGCTGGCG

P_k AAGAGCCTCG CGCGCTTCTG CCTGCGCAAC CAGGT-GCAG ACCTTCATCG TCAGCTGGCG

P_c TCAGCTGGCG CAACCCCGAT GTCCG-GCAT CGCGAATGGG GCCTGTCGAG TTATGTCGAG

P_p2 AACAGCTTCG TGCAGTACAT GCTCAAGAAC GGCTT-GCAG GTGTTCATGA TCAGTTGGCG

771

P_a CAACCC-GAC CAAGGCCCAG CGCGAATGGG GCCTGTCGAC CTACATCGAT GCGCTCAAGG

P_e CAACCC-CAC CAAGGAACAG CGCGAGTGGG GCCTGTCGAC TTATATCGAA GCGCTCAAGG

P_f CAACCC-GAC CAAGGCCCAG CGCGAATGGG GCCTGTCGAC CTATATCGAG GCGCTCAAGA

P_m CAACCC-GAC CAAGGCGCAG CGCGAGTGGG GCCTGTCCAC CTACATCGAG GCGCTCAAGG

P_n CAACCC-AAC CAAGGCGCAG CGCGAGTGGG GCCTGTCCAC CTACATCGAG GCGCTCAAGG

P_r CAACCC-GAC CAAGGCGCAG CGCGAGTGGG GCCTGTCCAC CTACATCGAG GCCCTCAAGG

P_s CAACCC-GAC CAAGGCGCAG CGCGAGTGGG GCCTGTCCAC CTATATCGCG GCGCTCAAGG

P_sp CAACCC-GAC CAAGGCGCAG CGCGAATGGG GCCTGTCCAC CTACATCGAG GCGCTCAAGG

P_p1 CAACCC-GAC CAAGGCGCAG CGTGAGTGGG GCCTGTCCAC CTACATCGAG GCGCTCAAGG

P_d CAACCC-GAC CAAGGCGCAA CGCGAGTGGG GCCTGACCAC CTACATCGAG GCCCTCAAGG

P_k CAACCC-GAC CAAGGCACAG CGTGAGTGGG GCCTGACCAC TTACATCGAG GCGCTGAAGG

P_c CAACCCCGAT GTCCG-GCAT CGCGAATGGG GCCTGTCGAG TTATGTCGAG GCCGTGGAAG

P_p2 CAACCCCGAC CCGCG-CCAC CGCGAATGGG GCCTGTCCAC GTACGTACAA GCCCTGGAAG

831

P_a AAGCCGTCGA CGTGATCCTG GCGATCACCG GCAGCAAAGA CCTGAACATG CTCGGCGCCT

P_e AAGCGGTCGA TGTGGTCACG GCGATTACCG GCAGCAAGGA CGTCAACATG CTCGGCGCCT

P_f AGGCCGTTGA GGTAGTCTGC GCGATCACCG GCAGTGCTGA TGTGAACGTA CTCGGCGCCT

P_m AAGCCATCGA CGTCATCTGC GCCATCACCG GCAGCAAAGA CGTGAACATG CTCGGCGCCT

P_n AAGCCATCGA CGTCATCTGC GCCATCACCG GCAGCAAGGA CATCAACATG CTCGGCGCCT

P_r AAACCATCGA TGTCATCCTG AAGATCACCG GCGCGAAGGA CCTCAACATC CTCGGCGCCT

P_s AAGCCATCGA GGTCATCTGC GCCATCACCG GCAGCAAGGA CGTCAACATG CTCGGCGCCT

P_sp AAGCCATCGA CGTGATCTGC GCCATCACCG GCAGCAAGGA GGTCAACATG CTTGGCGCCT

P_p1 AAGCCATCGA CGTGATCTGC GCCATCACCG GCAGCAAGGA CGTCAACATG CTCGGCGCCT

P_d AAGCCATCGA CGTGGTCCTG GCGATCACCG GCAGCAAGGA CCTGAACATG CTCGGCGCCT

P_k AAGCCATCGA CGTGGTCCTC GCCATCACCG GCGCCAAGGA CCTGAACATG CTCGGCGCCT

P_c AGGCCATGAA TGTCTGCCGG GCGATCACCG GCAGCCGCGA CGTGAACCTC ATGGGCGCCT

P_p2 AAGCGCTGAA CGCCTGCCGC AGCATCAGCG GCAACCGCGA CCCCAACCTG ATGGGTGCCT

891

P_a GCTCCGGCGG CATCACCTGC ACCGCCCTGC TCGGTCACTA CGCAGCGCT- -GGGCGA--A

P_e GCTCCGGCGG CATCACCTGC ACCGCGCTGC TGGGCCACTA CGCGGCGCT- -GGGGGA--G

P_f GCTCCGGCGG CGCCACCACC GCCTCGCTGC TCGGCCACTA CGCAGCCAT- -CGGCGA--A

P_m GCTCCGGTGG CCTGACCACT GCTTCGCTGC TCGGCCACTA CGCCGCGCT- -CGGCCA--A

P_n GCTCCGGCGG CCTGACCACC GCATCGCTGC TCGGCCACTA CGCCGCGCT- -CGGCCA--G

P_r GCTCCGGCGG CATCACCACC GTCGCCCTGC TCGGCCACTA CCAGGCCAC- -CGGCCA--G

P_s GCTCCGGCGG CCTGACCACC GCCTCCCTGC TCGGCCACTA CGCCGCCCT- -CGGCGA--G

P_sp GTTCCGGCGG CCTGACCACT GCCTCGCTGC TCGGTCACTA TGCAGCGCT- -TGGCCA--G

P_p1 GCTCCGGCGG CCTGACCACG GCCTCGCTGC TCGGTCACTA TGCAGCGCT- -TGGCCA--G

P_d GCTCCGGCGG CATCACCACG GTGACGCTGC TGGGCCACTA CGCGGCCCT- -GGGCGA--G

P_k GCTCCGGCGG CATCACCACC GTGACCCTGC TGGGCCACTA CGCCGCGCT- -GGGCGA--G

P_c GCGCCGGCGG CCTGACCATC GCCGCGCTGC AAGGCCACCT -GCAGGCCAA GCGGCAATTG

P_p2 GCGCCGGCGG CCTGACCATG GCTGCGCTGC AGGGCCACCT -GAAGGCCAA ACATCAGTTG

951

P_a ACCAAGGTCA ATGCCCTGAC CCTGCTGGTC AGCGTCCTCG ACACCACCCT CGACAACCAG

P_e AAGAAGGTCA ACGCCCTGAC CCTGCTGGTG AGCGTGCTGG ACACCACCCT CGACACCCAG

P_f CAGAAAGTGC ATGCCCAGAC GCTGCTGGTC AGCGTGCTGG ATACTCAGCT GGACAGCCAG

P_m CCTAAAGTCA ATGCCCTGAC CTTGCTGGTC AGCGTGCTCG ACACCCAGCT CGACACCCAG

P_n CCGAAGGTCA ATGCCCTGAC CCTGCTGGTC AGCGTGCTCG ACACCCAGCT AGACACTCAG

P_r AACAAGGTCA ACGCCTTCAC CCAGATGGTC AGCGTGCTCG ACTTCAACCT CAACACCCAG

P_s CAGAAGGTGC ATGCCCTGAC CCTGCTGGTC AGCGTGCTCG ACACCCAGCT CGACACCCAG

P_sp CAGAAGGTCA ACGCCCTGAC CCTGCTGGTC AGCGTGCTCG ACACCCAGCT CGACACCCAG

P_p1 CAGAAGGTCA ACGCCCTGAC CCTGCTGGTC AGCGTGCTCG ACACTCAGCT AGACACCCAG

P_d AACAAGGTGC ATGCCTTCAC CCAGATGGTC AGCGTGCTGG ACTTCGAGAT GAACACCCAG

P_k GAGAAGGTCC ACGCCTTCAC CCAGATGGTC AGCGTGCTCG ACTTCGAGCT CGACACCACC

P_c CGCCGGGTGT TCAGTTCCAC CTATCTGGTC AGCTTGCTCG ACAGCCAGCT GGACAGCCCG

P_p2 CGCAAGATCC GCAGCGCCAC GTACCTGGTC AGCCTGCTCG ACAGCAAGTT CGATAGCCCC

1011

P_a GTCGCACTGT TCGTCGACGA ACAGACCCTG GAA-ACCGCC AAGCGCCACT CCTATCAGGC

P_e GTCGCGCTGT TCGTCGACGA GCAGACCCTG GAA-GCGGCC AAGCGCCACT CCTACCAGGC

P_f TACGCGCTGT TCGCCGACGA GCAGACCCTG GAA-GCGGCC AAACGCCGTT CCTACCAGGC

P_m GTAGCGCTGT TCGCCGACGA GAAGACCCTG GAA-GCGGCC AAGCGCCGCT CCTACCAGGC

P_n GTAGCACTGT TCGCCGACGA GAAGACTCTG GAA-GCCGCC AAGCGTCGCT CCTACCAGGC

P_r GTCGCCCTGT TCGCCGACGA ACAGACCCTG GAG-GCCGCC AAGCGCCGGT CGTACCAGGC

P_s GTGGCGCTGT TCGCCGACGA GAAGACCCTC GAG-GCCGCC AAGCGCCGTT CCTACCAGGC

P_sp GTTGCCCTGT TCGCCGACGA GAAGACCCTG GAA-GCCGCC AAACGCCGCT CCTACCAGGC

P_p1 GTTGCCCTGT TCGCCGACGA GAAGACCCTG GAA-GCCGCC AAACGCCGCT CCTACCAGGC

P_d ATCGCCCTGT TCGCCGACGA GAAGACCCTG GAG-ACCGCC AAGCGCCGCT CGTACCAGTC

P_k ATCGCCCTGT TCGCCGACGA GAAGACCCTC GAG-GCCGCC AAGCGCCGCT CCTACCAATC

P_c GCGACCCTGT TCGTCGACGA GCAGACCCTG GAG-GCGGCC AAGCGTCGCT CCTATCAGCA

P_p2 GCCAGCCTGT TCGCCGACGA GCAGACGGTC GAG-GCCGCC AAGCGCCGCT CCTATCAACG

1071

P_a CGGGGTGCTT GAAGGCCGCG ACATGGCCAA GGTGTTTGCC TGGATGCGCC CTAACGACCT

P_e CGGCGTGCTG GAAGGTCGCG ACATGGCCAA GGTGTTTGCC TGGATGCGCC CCAACGACCT

P_f CGGTGTGCTG GAAGGCAGCG ACATGGCCAA GGTGTTCGCC TGGATGCGCC CCAACGACCT

P_m CGGAGTGCTG GAAGGCAGCG ACATGGCCAA GGTTTTCGCC TGGATGCGCC CCAACGACCT

P_n CGGCGTGCTG GAAGGCAGCG ACATGGCCAA GGTGTTCGCC TGGATGCGCC CCAATGACCT

P_r CGGCGTGCTG GAGGGCAAGG ACATGGCCAA GGTGTTCGCC TGGATGCGGC CGAACGACCT

P_s CGGCGTGCTG GAAGGCAGCG ACATGGCCAA GGTGTTCGCC TGGATGCGCC CGAATGACCT

P_sp CGGCGTACTG GAGGGTAGCG ACATGGCCAA GGTGTTCGCC TGGATGCGCC CCAACGACCT

P_p1 CGGCGTACTG GAGGGTAGTG ACATGGCCAA GGTGTTCGCC TGGATGCGCC CCAACGACCT

P_d CGGCGTGCTG GAAGGCAAGG ACATGGCCAA GGTGTTCGCC TGGATGCGGC CGAACGACCT

P_k CGGCGTGCTG GAAGGCAAGG ACATGGCCAA GGTGTTCGCC TGGATGCGGC CGAACGACCT

P_c GGGCGTGCTC GACGGTCGCG ACATGGCCAA GGTGTTCGCC TGGATGCGGC CCAACGACCT

P_p2 GGGTGTGCTG GACGGCGGCG AGGTGGCGAG GATTTTCGCC TGGATGCGCC CCAACGACCT

1131

P_a GATCTGGAAC TACTGGGTCA ACAACTACCT GCTCGGCAAC GAGCCGCCGG TGTTCGACAT

P_e GATCTGGAAC TACTGGGTCA ATAACTACCT GCTGGGCAAC GAGCCGCCGG TGTTCGACAT

P_f GATCTGGAAT TACTGGGTCA ACAACTACCT GCTCGGCAAC GAGCCGCCGG TGTTCGACAT

P_m GATCTGGAAC TACTGGGTCA ACAACTACCT GCTCGGCAAC GAGCCGCCGG TGTTCGACAT

P_n GATCTGGAAC TACTGGGTCA ACAACTACCT GCTGGGCAAC GAGCCGCCGG TGTTCGACAT

P_r GATCTGGAAC TACTGGGTCA ACAACTACCT GCTGGGCAAC GAGCCGCCGG CCTTCGACAT

P_s GATCTGGAAC TACTGGGTCA ACAACTACCT GCTCGGCAAC GAGCCGCCGG TGTTCGACAT

P_sp GATCTGGAAC TACTGGGTCA ACAACTACCT GCTCGGCAAC GAGCCGCCGG TGTTCGACAT

P_p1 GATCTGGAAC TACTGGGTCA ACAACTACCT GCTCGGCAAC GAGCCACCGG TGTTCGACAT

P_d GATCTGGAAC TACTGGGTCA ACAACTACCT GCTGGGCAAC GAGCCGCCGG CCTTCGACAT

P_k GATCTGGAAC TACTGGGTCA ACAACTACCT GCTCGGCAAC GAGCCGCCGG CCTTCGACAT

P_c GATCTGGAAT TACTGGGTCA ACAATTACCT GCTGGGCAAG GAGCCGCCGG CGTTCGACAT

P_p2 GATCTGGAAC TACTGGGTCA ACAACTACCT GCTTGGCAAA GCCCCGCCCG CGTTCGACAT

**PhaC1R2 5’ TGGTGTCGTTGTTCCAG 3’**

1191

P_a CCTGTTCTGG AACAACGACA CCACGCGCCT GCCGGCCGCC TTCCATGGCG ACCTGATCGA

P_e CCTGTTCTGG AACAACGACA CCACGCGCTT GCCGGCCGCC TTCCATGGCG ACCTGATCGA

P_f TCTTTACTGG AACAACGACA CCACCCGCCT GCCCGCCGCC TTGCACGGCG AGTTCATCGA

P_m TCTCTACTGG AACAACGACA CCACACGCCT GCCGGCTGCG CTGCACGGCG AGTTCATCGA

P_n CCTCTACTGG AACAACGACA CCACACGCCT GCCCGCCGCG CTGCACGGCG AATTCATCGA

P_r CCTCTACTGG AACAACGACA CCACGCGCCT GCCCGCGGCG CTGCACGGTG AGCTTGTGGA

P_s CCTCTACTGG AACAACGACA CCACGCGCCT GCCGGCCGCC CTGCACGGCG AGTTCATCGA

P_sp CCTTTATTGG AACAACGACA CCACACGTTT GCCGGCCGCG CTACACGGCG AGTTCATCGA

P_p1 CCTCTACTGG AACAACGACA CCACGCGCCT GCCGGCCGCG CTGCATGGCG AGTTCATCGA

P_d CCTGTTCTGG AACAACGACA CCACCCGCCT GCCGGCCGCG CTGCACGGCG AACTGGTGGA

P_k CCTGTTCTGG AACAACGACA CCACGCGCCT GCCCGCCGCG CTGCACGGCG AGCTGGTGGA

P_c CCTCTACTGG AACAACGACA ACACCCGGCT GCCGGCGGCC TTCCACGGCG ACCTGCTGGA

P_p2 CCTCTACTGG AACGCCGACA ACACACGCCT GCCGGCAGCA CTGCACGGCG ACTTGCTGGA

1251

P_a AATGTTCAAA AGTAATCCAC TGACCCGTGC CGATGCACTG GAAGTGTGCG GTACGCCGAT

P_e GCTGTTCAAG AACAACCCGC TGGTGCGCGC CAACGCCCTG GAGGTGTGCG GCACGCCCAT

P_f GATGTTCCAG ACCAACCCAC TGACCCGCGC AGGCGCGCTG GAAGTCTGCG GCACGCCCAT

P_m AATGTTCCAG ACCAACCCGT TGACCCGTCC CGGCGCGCTG GAAGTGTGCG GTACGCCGAT

P_n AATGTTCCAG ACCAACCCGC TGACCCGCCC GGGCGCGCTG GAGGTGTGCG GCACACCGAT

P_r GATGTTCAAG ACCAACCCGC TGACCCGCCC CCACGCCCTG GAAGTCAGCG GCACCCCCAT

P_s GATGTTCCAG ACCAACCCGC TGACCCGCCC GGGCGCGCTG GAGGTCTGCG GCACGCCGAT

P_sp CATGTTCCAG ACCAACCCAC TGACCCGTCC GGGCGCGCTG GAAGTCTGCG GCACACCGAT

P_p1 GATGTTCCGG ACCAACCCAC TGACCCGCCC GGGCGCGCTG GAAGTCTGCG GCACACCGAT

P_d GATGTTCAAG ACCAATCCGC TGACCCGCGC GGGCGCGCTG GAAGTCTCCG GCACGCCGAT

P_k AATGTTCAAG AGCAACCCGC TCAGCCGCCC CGGCGCGCTG GAGGTCGCCG GCACCCCCAT

P_c CTTCTTCAAG CACAATCCGC TGGGCCATGC GGGTGGCCTG GAAGTCTGCG GCACGCCCAT

P_p2 CTTTTTCAAG CACAACCCGC TCACCCACCC GGCAGGCCTC GAAGTGTGCG GCACGCCCAT

1311

P_a CGACCTGAAG AAAGTCACCG CCGACATCTA CAGCCTGGCC GGCACCACCG ATCACATCAC

P_e CGACCTCAAG CAGATCACCG CCGATATTTA CTCACTGGCG GGCACCAACG ACCACATCAC

P_f CGACCTCAGT CAGGTCACCT GTGACTACTA CGTGGTGGCC GGCAGCAACG ACCACATCAC

P_m CGACCTCAAA CAGGTCACCT GCGACTTCTT CGTCGTCGCC GGCACCACCG ACCACATCAC

P_n CGACCTCAAG CAGGTGACCT GCGATTTCTT CGTCGTCGCC GGCACCACCG ACCACATCAC

P_r GGCAACCCCA AGGCGCGCTT CTCCACCAGC AGCGACATGC C--GGAGGAT CCGAAGCTCT

P_s CGACCTCAAG CAGGTCACCT GCGACTTCTT CTGCGTCGCC GGCACCACCG ACCACATCAC

P_sp CGACCTGAAG CAGGTCACCT GTGATTTCTT CGTCGTCGCT GGCACCACCG ACCACATCAC

P_p1 CGACCTCAAG CAGGTCACCT GTGATTTCTT CGTCGTCGCT GGCACCACCG ACCACATCAC

P_d CGACCTCAAG CAGGTCACCT GCGACTTCTA CTGCGTGGCC GGCCTGTCCG ACCACATCAC

P_k CGACCTCAAG CAGGTCAAAT GCGACTTCTA TTGCCTGGCC GGACTGACCG ACCACATCAC

P_c CGATCTGCAG AAGGTCACGG TCGACAGCTT CAGCGTCGCC GGGATCAACG ATCACATCAC

P_p2 CGACCTCAAG CAGGTCGACC TGGACAGTTT TACCGTGGCC GGCAGCAACG ATCACATCAC

1371

P_a CCGACATCTA CAGCCTGGCC GGCACCACCG ATCACATCAC TCCGTGGCAG TCCTGCTACC

P_e CCGATATTTA CTCACTGGCG GGCACCAACG ACCACATCAC GCCGTGGCAA TCCTGCTACA

P_f GTGACTACTA CGTGGTGGCC GGCAGCAACG ACCACATCAC GCCCTGGACG TCCTGCTACA

P_m GCGACTTCTT CGTCGTCGCC GGCACCACCG ACCACATCAC GCCATGGGAT TCCTGCTACA

P_n GCGATTTCTT CGTCGTCGCC GGCACCACCG ACCACATCAC GCCCTGGGAT TCCTGCTACA

P_r GCGACTTCTA CTGCGTGGCC GGCACCACCG ACCACATCAC CCCCTGGGAA GCCTGCTACC

P_s GCGACTTCTT CTGCGTCGCC GGCACCACCG ACCACATCAC GCCCTGGGAT TCCTGCTACA

P_sp GTGATTTCTT CGTCGTCGCT GGCACCACCG ACCACATCAC CCCCTGGGAC TCCTGCTACA

P_p1 GTGATTTCTT CGTCGTCGCT GGCACCACCG ACCACATCAC CCCCTGGGAT TCCTGCTACA

P_d GCGACTTCTA CTGCGTGGCC GGCCTGTCCG ACCACATCAC GCCCTGGGAA GCCTGCTACC

P_k GCGACTTCTA TTGCCTGGCC GGACTGACCG ACCACATCAC GCCCTGGGAA GCCTGCTATC

P_c TCGACAGCTT CAGCGTCGCC GGGATCAACG ATCACATCAC CCCCTGGGAC GCGGTCTATC

P_p2 TGGACAGTTT TACCGTGGCC GGCAGCAACG ATCACATCAC CCCGTGGGAC GCCGTGTATC

1431

P_a GTTCGGCGCA GCTGTTTGGC GGCAATGTCG AGTTCGTGCT GTCCAACAGC GGGCATATCC

P_e AGTCGGCGCA GCTGTTTGGC GGCAAGGTGG AATTCGTGTT GTCCAGCAGC GGGCATATCC

P_f AGTCGGCGCG GCTGTTCGGC GGCCAGTGCG AGTTCGTGCT GTCCAGCAGC GGGCACATCC

P_m AATCGGCTCA TCTGTTCGGC GGCAAATGCG AGTTCGTGCT CTCCAACAGC GGCCATATCC

P_n AGTCGGCTCA TCTGTTCGGC GGCAAGTGCG AATTCGTACT GTCCAACAGC GGACATATCC

P_r GCTCGGCGCG GCTGCTGGGC GGCAAGTGCG AGTTCGTGCT GTCCAACAGC GGCCATATCC

P_s AGTCGGCCCA CCTGTTCGGC GGCAAGTGCG AGTTCGTGCT GTCCAACAGC GGCCATATCC

P_sp CCTGTTCGGT GGTAAGTGTG AGTTCGTGCT GTCCAACAGC GGCCATATCC AGAGCATCCT

P_p1 AGTCGGCGCA CCTGTTCGGC GGCAAGTGCG AGTTCGTGCT GTCCAACAGC GGCCATATCC

P_d GCTCCGCGCG GCTGCTGGGC GGCAAGTGTG AGTTCATCCT CTCCAACAGC GGGCACATCC

P_k GCTCCGCCCG CCTGCTTGGC GGCAAGTGCG AGTTCATCCT CTCCAACAGC GGGCACATCC

P_c GTTCGACCCT GCTGCTGGGC GGCGACAAGC GTTTCGTGCT GTCCAACAGC GGGCATGTGC

P_p2 GCTCGGCCTT GCTGCTCGGT GGCGACCGGC GTTTCGTGCT GGCCAACAGC GGGCATATTC

**PhaC1R1 5’ CGGGTTGAGRATGCTCTG 3’**

1491

P_a AGAGCATCCT CAACCCGCCG GGCAACCCCA

P_e AGAGCATCCT CAACCCGCCG GGCAACCCCA

P_f AGAGCATCCT CAACCCGCCG GGCAACCCCA

P_m AGAGCATTCT CAACCCGCCG GGCAACCCCA

P_n AAAGCATTCT CAACCCGCCA GGCAACCCCA

P_r AGAGCATCCT CAACCCGCCG GGCAACCCCA

P_s AGAGCATCCT CAACCCGCCG GGCAACCCCA

P_sp AGTCGGCGCA CAACCCGCCG GGCAATCCCA

P_p1 AGAGCATTCT CAACCCGCCG GGCAACCCCA

P_d AGAGCATCCT CAACCCGCCG GGCAACCCCA

P_k AGAGCATCCT CAACCCACCG GGCAACCCCA

P_c AGAGCATCCT CAACCCGCCG GGCAACCCCA

P_p2 AAAGCATCAT CAACCCACCG GGCAACCCCA
