# Supplementary figures and images for: Screening of polyhydroxyalkanoate-producing bacteria and PhaC-encoding genes in two hypersaline microbial mats from Guerrero Negro, Baja California Sur, Mexico
Source: PeerJ. 2018 May 7;6:e4780. doi: 10.7717/peerj.4780 (PMC5944434; doi:10.7717/peerj.4780)

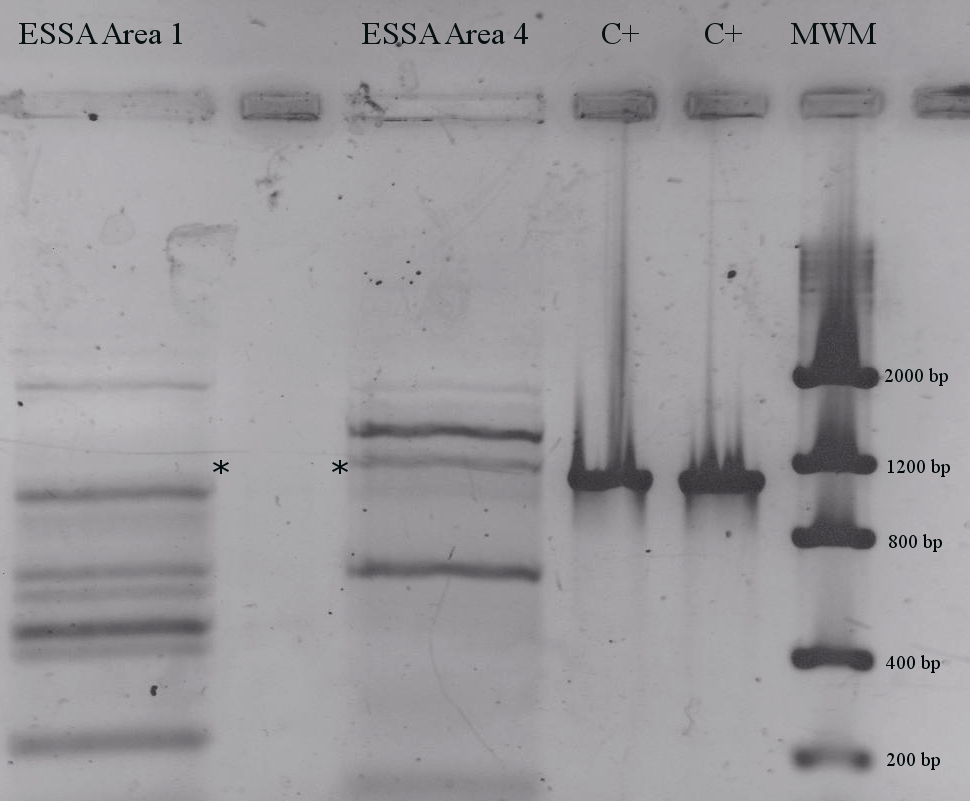

Supplement: Supplemental Information 2 — phaC1 amplification from environmental DNA of microbial mats using primers phaC1F2 and phaC1R1 in the first round of nested-PCR. MWM= molecular weight marker DNA Low Mass Ladder from Invitrogen (15628-050); C+= positive control with DNA of KT2440 strain. The asterisks show the bands that were excised and purified. [file peerj-06-4780-s002.png]

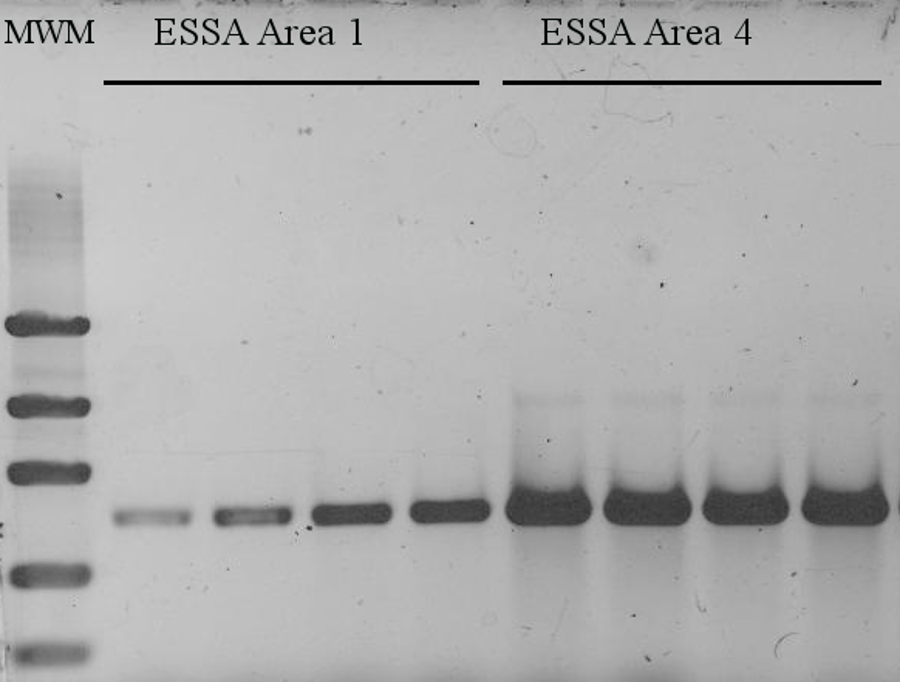

Supplement: Supplemental Information 3 — Quadruplicates of phaC1 amplification from environmental DNA of microbial mats using primers phaC1F1 and phaC1R2 in the second round of nested-PCR. MWM = molecular weight marker DNA Low Mass Ladder from Invitrogen (15628-050). [file peerj-06-4780-s003.png]
