# Supplementary material for: Screening of polyhydroxyalkanoate-producing bacteria and PhaC-encoding genes in two hypersaline microbial mats from Guerrero Negro, Baja California Sur, Mexico
Source: PeerJ. 2018 May 7;6:e4780. doi: 10.7717/peerj.4780 (PMC5944434; doi:10.7717/peerj.4780)
Supplement: Supplemental Information 4 — Closest identities of deduced-amino acid sequences, inferred from phaC and phaC1 genes retrieved from hypersaline microbial mats from ESSA A1 and ESSA A4. [file peerj-06-4780-s004.docx]

| Clone number | Site | PhaC class | Closest identity/Accession number at GenBank | Identity value (%) |
| --- | --- | --- | --- | --- |
| 1 | ESSA A1 | I | PHA synthase class I, *Woodsholea maritima*/ WP019961734 | 82 |
| 2 | ESSA A1 | I | PHA synthase, *Marinobacter* sp. DSM 26671/ SFE82661 | 97 |
| 4 | ESSA A1 | I | PHA synthase class I, *Marivita hallyeonensis/* WP072776758 | 94 |
| 5 | ESSA A1 | I | PHA synthase, *Marinobacter* sp. DSM 26671/ SFE82661 | 99 |
| 7 | ESSA A1 | I | PHA synthase class I, *Tistlia consotensis*/ WP085122734 | 80 |
| 8 | ESSA A1 | I | PHA synthase class I, *Oceanicaulis* sp. HL-87/ WP081891145 | 82 |
| 11 | ESSA A1 | I | PHA synthase class I, *Marivita hallyeonensis* DPG-28 */* WP072776758 | 96 |
| 13 | ESSA A1 | I | PHA synthase class I, *Rubrimonas cliftonensis*/ WP093247837 | 68 |
| 14 | ESSA A1 | I | PHA synthase class I, *Granulosicoccus antarcticus/* WP088921818 | 69 |
| 16 | ESSA A1 | I | PHA synthase, *Hyphomonas* sp. BRH_c22/ KJS39743 | 77 |
| 17 | ESSA A1 | I | PHA synthase class I, *Granulosicoccus antarcticus/* WP088921818 | 68 |
| 18 | ESSA A1 | I | PHA synthase class I, Rhodobacteraceae bacterium/ WP084860574 | 91 |
| 21 | ESSA A1 | I | PHA synthase, *Gemmobacter aquatilis,* SEN82362 | 80 |
| 23 | ESSA A1 | I | PHA synthase, *Rubrimonas cliftonensis*/ SEB00608 | 85 |
| 56 | ESSA A1 | I | PHA synthase class I, *Cobetia amphilecti,* WP064503336 | 98 |
| 59 | ESSA A1 | I | PHA synthase class I, *Rubritepida flocculans*/ WP027284337 | 75 |
| 71 | ESSA A4 | I | PHA synthase class I, *Citreimonas salinaria,* WP089885123 | 87 |
| 79 | ESSA A4 | I | PHA synthase class I, *Magnetospirillium sp*/ WP096703591 | 76 |
| 98 | ESSA A4 | I | PHA synthase class I, *Acidisphaera rubrifaciens,* WP084623775 | 69 |
| 107 | ESSA A4 | I | PHA synthase class I, *Henriciella marina*/ WP018147140 | 73 |
| 123 | ESSA A4 | I | PHA synthase, uncultured bacterium/ALB01203 | 67 |
| 131 | ESSA A4 | I | PHA synthase class I, *Azospirillum oryzae,* WP085084697 | 80 |
| 143,145,146,159,191 | ESSA  A1 | II | PHA synthase class II, *Pseudomonas putida,*  WP070384427 | 99 |
| 194, 197, 202, 210, 217, 218, 223, 237 | ESSA  A4 | II | PHA synthase class II, *Pseudomonas putida,* WP070384427 | 99 |
