# Supplementary material for: Screening of polyhydroxyalkanoate-producing bacteria and PhaC-encoding genes in two hypersaline microbial mats from Guerrero Negro, Baja California Sur, Mexico
Source: PeerJ. 2018 May 7;6:e4780. doi: 10.7717/peerj.4780 (PMC5944434; doi:10.7717/peerj.4780)
Supplement: Supplemental Information 5 — Clustering with CD-HIT of putative-PhaC sequences retrieved from clone libraries derived of the environmental DNA isolated from microbial mats of ESSA A1 and ESSA A4. A threshold value of 97% was employed. [file peerj-06-4780-s005.docx]

| Cluster | PhaC class | Number of sequences | Representative clone | Site |
| --- | --- | --- | --- | --- |
| 1 | I | 1 | 59 | ESSA A1 |
| 2 | I | 1 | 7 | ESSA A1 |
| 3 | I | 1 | 16 | ESSA A1 |
| 4 | I | 1 | 123 | ESSA A4 |
| 5 | I | 1 | 8 | ESSA A1 |
| 6 | I | 2 | 21 | ESSA A1 |
| 7 | I | 1 | 1 | ESSA A1 |
| 8 | I | 1 | 56 | ESSA A1 |
| 9 | I | 1 | 98 | ESSA A4 |
| 10 | I | 1 | 23 | ESSA A1 |
| 11 | I | 3 | 4 | ESSA A1 |
| 12 | I | 1 | 107 | ESSA A4 |
| 13 | I | 1 | 2 | ESSA A1 |
| 14 | I | 2 | 5 | ESSA A1 |
| 15 | I | 2 | 79 | ESSA A4 |
| 16 | I | 2 | 14 | ESSA A1 |
| 17 | I | 1 | 71 | ESSA A4 |
| 18 | I | 1 | 131 | ESSA A4 |
| 19 | I | 1 | 18 | ESSA A1 |
| 20 | I | 1 | 13 | ESSA A1 |
| 21 | II* | 32 | 223 | ESSA A1, ESSA A4 |

*Unique cluster of PhaC Class II.
